# Effects of Different Stressors Are Modulated by Different Neurobiological Systems: The Role of GABA-A Versus CB1 Receptor Gene Variants in Anxiety and Depression

**DOI:** 10.3389/fncel.2019.00138

**Published:** 2019-04-09

**Authors:** Xenia Gonda, Peter Petschner, Nora Eszlari, Sara Sutori, Zsofia Gal, Szabolcs Koncz, Ian M. Anderson, Bill Deakin, Gabriella Juhasz, Gyorgy Bagdy

**Affiliations:** ^1^Department of Psychiatry and Psychotherapy, Semmelweis University, Budapest, Hungary; ^2^MTA-SE Neuropsychopharmacology and Neurochemistry Research Group, Hungarian Academy of Sciences, Semmelweis University, Budapest, Hungary; ^3^NAP-2-SE New Antidepressant Target Research Group, Hungarian Brain Research Program, Semmelweis University, Budapest, Hungary; ^4^Department of Pharmacodynamics, Faculty of Pharmacy, Semmelweis University, Budapest, Hungary; ^5^Neuroscience and Psychiatry Unit, Division of Neuroscience and Experimental Psychology, School of Biological Sciences, Faculty of Biological, Medical and Human Sciences, Manchester Academic Health Sciences Centre, The University of Manchester, Manchester, United Kingdom; ^6^Greater Manchester Mental Health NHS Foundation Trust, The University of Manchester, Manchester, United Kingdom; ^7^SE-NAP 2 Genetic Brain Imaging Migraine Research Group, Semmelweis University, Budapest, Hungary

**Keywords:** types of stress, depression, anxiety, gene-environment interaction, GABA, *GABRA6*, *CNR1*, endocannabinoid system

## Abstract

Environmental stress and its interaction with genetic variation are key contributors in the development of depression and anxiety, yet there is a failure to identify replicable genetic variants and gene-interaction effects in the background of these psychiatric symptoms. Recently it has been reported that *5-HTTLPR* and NOSI interact with financial but not other types of recent stressors in the development of depression. In the present study we investigated the interaction of *GABRA6* rs3219151 and *CNR1* rs7766029 in interaction with different types of recent life events on the presence of depression and anxiety in a large general population sample. 2191 participants completed the List of Threatening Experiences questionnaire which covers four categories of stressful life events (financial problems, illness/personal problems, intimate relationships, and social network) experienced over the previous year and the Brief Symptom Inventory for depression and anxiety symptoms. Participants were genotyped for rs3219151 and rs7766029. Data were analyzed with linear regression models with age and gender as covariates. Results indicated that *CNR1* rs7766029 interacted significantly with financial but not other types of life events both in case of depression and anxiety symptoms. In contrast, *GABRA6* rs3219151 showed a significant interaction with social network related life events in case of anxiety and with illness/personal problem-related life events in case of depression. Our results suggest that the psychological impact of different types of recent stress may be differentially modulated by distinct molecular genetic pathways. Furthermore, in case of certain genetic variants, the occurring psychiatric symptom may depend on the type of stress experienced.

## Introduction

In spite of the ever-increasing interest in genetic mechanisms of depression, findings in candidate gene association studies have generally not been replicated and not detected in genome-wide association studies (Flint and Kendler, 2014; Dunn et al., 2015; Gonda et al., 2018c). Recently, a study investigating variation in 7 genes in pathways previously implicated in the neurobiology of depression found that none influenced depressive phenotype in the absence of exposure to recent stress (Gonda et al., 2018b). However, in those exposed to moderate and/or severe stress, the majority of variants showed “relevance,” a Bayesian measure, to phenotype. In some genes relevance was greatest for moderate rather than severe stress suggesting that may recruit different or additional genes and/or neurotransmitter systems. The *5-HTTLPR* polymorphism is the most widely studied candidate mechanism of depression with the most contradictory findings in individual (Juhasz et al., 2015) and meta-analytic studies (Bleys et al., 2018; Culverhouse et al., 2018a,b), We recently reported that the polymorphism did not modify risk of depression either alone or in interaction with a unitary measure of life events (Gonda et al., 2016a,b, 2018a). However, the short allele of *5-HTTLPR* was associated with increased depressive symptoms in those exposed to recent financial stressors, but not in those exposed to any other types of recent life events. Furthermore, in those aged 30 or younger the short allele showed an inverse impact on depression when exposed to social stress, reflecting a possible protective effect of this variant. These results suggest that the effect of the majority of genes involved in depression may be manifested only under certain environmental settings, and different types of stressors can exert their effects via divergent genetic and ultimately neurochemical pathways. Consequentially, different types of stressors should be analyzed separately in gene-environment interaction studies of depression.

Our currently used antidepressants (Gonda et al., 2018c), all of which – with the current exception of agomelatine and the possible exception of esketamine – act primarily on monoaminergic systems show only a frustratingly limited clinical efficacy. Therefore it is crucial to understand the involvement of other genes and neurochemical systems, and especially in interaction with possibly pro-depressive stress in the background of depression.

Recently, we reported that a variation of the *GABRA6* gene encoding the alpha6 subunit of the GABA-A receptor has no main effect on depression but exerts a significant impact following exposure to recent stress on several depression- and anxiety-related symptoms, giving rise to a symptom constellation that significantly increases suicide risk (Gonda et al., 2017). In this study the different types of recent life stressors were not studied separately, nevertheless, according to previous studies recent social stress may be mediated by the *GABRA6* gene (Uhart et al., 2004). Another system involved both in stress and in depression and anxiety, is the endocannabinoid system, with its relevance underlined by the withdrawal of the endocannabionoid-1 receptor antagonist rimonabant from the market due to severe psychological side effects including increased anxiety and depression (Christensen et al., 2008). While previously a significant interaction effect between stress and *CNR1* receptor variation on depressive symptoms (Juhasz et al., 2009) has been reported, and an effect of *CNR1* receptor variants on anxiety influenced also by serotonin receptor variants have been described (Lazary et al., 2009, 2011) the role of different stress types in this interaction has not been studied. Thus, the aim of the present study was to investigate the effect of the interaction between different types of recent life events and *GABRA6* or *CNR1* gene variants on recent depressive/anxiety symptoms in a large European general population sample.

## Materials and Methods

### Study Population

Under the aegis of the NewMood study (New Molecules in Mood Disorders, LHSM-CT-2004-503474, Sixth Framework Program of the European Union) (Deakin et al., 2011), adult subjects were recruited from the general population through advertisements, a website and general practices. 2269 subjects (923 in Budapest, Hungary, and 1346 in Manchester, United Kingdom) provided self-reported data on gender, age, recent stress, current depression, and anxiety scores by filling out a questionnaire pack, and provided genetic data by a saliva sampling kit. All of them reported to be of European white ethnic origin, and none of them reported to have any relative participating in the study.

All of the participants signed the official informed consent form. The study was carried out in accordance with the Declaration of Helsinki, and it was approved both by the Scientific and Research Ethics Committee of the Medical Research Council, Budapest, Hungary, and by the North Manchester Local Research Ethics Committee, Manchester, United Kingdom.

Among these participants, 2193 (*n* = 902 in Budapest, and *n* = 1291 in Manchester) were successfully genotyped for *CNR1* rs7766029, and *n* = 2206 (*n* = 902 in Budapest, and *n* = 1304 in Manchester) for *GABRA6* rs3219151.

### Phenotypes

The Brief Symptom Inventory (BSI) was used to measure current levels of anxiety and depression experienced within the past 7 days (Derogatis, 1993). Each depression and anxiety item was scored 0–4 depending on the distress caused. Anxiety score was calculated as the sum of anxiety symptom item scores divided by the number of completed items, and depression score was calculated as the sum of depression and additional item scores divided by the number of completed items.

Four different types of recent stress were measured by the List of Threatening Experiences (Brugha et al., 1985), summing the number of recent negative life events (RLEs) pertaining to each subscale, occurring within the last year. These subscales have already been used in gene-by-environment (GxE) interaction models in our previous genetic association analyses (Gonda et al., 2016a). The RLE-relationship subscale encompasses problems in marriage or steady relationship (e.g., “separation due to marital problems” and “broke off steady relationship”). The RLE-financial subscale denotes for financial crisis, becoming unemployed or unsuccessfully seeking work (e.g., “became unemployed or seeking work for more than 1 month” and “major financial crisis”). The RLE-illness/personal problems subscale embodies illness, injury, assault, problems with the police, a relative, a close friend or neighbor, court appearance, and losing or being stolen something (“serious illness,” “injury,” or “assault to self” and “serious problems with close friend,” “neighbor,” or “relative”). Finally, the RLE-social (social network disturbances) subscale comprises items on death, illness, injury or assault of a relative or friend (“close friend” or “other relative died” and “serious illness,” “injury,” or “assault to close relative”). Intercorrelations between the four subscales have been reported in Gonda et al. (2016a).

### Genotypes

Participants provided buccal mucosa cells collected by a cytology brush (Cytobrush plus C0012, Durbin PLC). Genomic DNA was extracted according to the protocol of Freeman et al. (2003). Genotyping was performed by the Sequenom’s MassARRAY technology (^1^San Diego, CA, United States) with the IplexTM assay. All laboratory work, carried out under the ISO 9001:2000 quality management requirements, was blinded regarding phenotypes.

### Statistical Analyses

IBM SPSS Statistics 21 was used to calculate descriptive statistics on the variables and their comparisons between the Budapest and Manchester subsamples, in addition, to run univariate general linear models for visualization purposes.

Plink v1.90^2^ was used to calculate Hardy-Weinberg equilibrium and minor allele frequency (MAF), and to build additive linear regression models on BSI anxiety and depression scores as primary and secondary analyses, and on each RLE subscale to test gene-environment correlation. Analyses were supported by scripts individually written in R 3.0.2 (R Core Team, 2013).

In the linear regression models run either in Plink or in SPSS, gender and age were always covariates. In case of the combined Budapest + Manchester sample, population was an additional covariate. In case of the GxE models on the BSI scores, main effects of both G and E were also included as covariates.

Only in order to facilitate visualization in the general linear models, RLE scores were divided into three categories as described previously (Gonda et al., 2016a): 0 event, 1 event, 2 or more events.

*P*-values of the primary tests were entered into QVALUE v1.0 (Storey et al., 2004) to calculate false discovery rate (FDR) *q*-values (without robust method), with the aim of correction for multiple testing. To estimate the proportion of true null hypotheses, tuning parameter lambda was set to be from 0 to 0.99 by 0.05, and a bootstrap method was used for automatically choosing lambda. In case of the primary tests, we consider results with a *q*-value ≤ 0.05 as significant.

In case of secondary tests and all descriptive statistics, we consider a *p*-value ≤ 0.05 as significant, and a *p*-value ≤ 0.10 as trend.

To evaluate statistical power of the primary tests, Quanto v1.2^3^ was used. Type I error rate was set to 0.05, and we assumed an R_GE_^2^ = 0.5% for the GxE term, an R_G_^2^ = 0% for the G term, and R_E_^2^ values, based on Pearson correlations and coefficients of determination (*n* = 2269), as the following. On BSI anxiety score, RLE-relationship has an R_E_^2^ of 0.0146, RLE-financial has an R_E_^2^ of 0.0562, RLE-illness/personal problems has an R_E_^2^ of 0.0324, and RLE-social has an insignificant R_E_^2^ of 0.0005. On BSI depression score, RLE-relationship has an R_E_^2^ of 0.0272, RLE-financial has an R_E_^2^ of 0.0655, RLE-illness/personal problems has an R_E_^2^ of 0.0396, and RLE-social has an insignificant R_E_^2^ of 0.0004. The MAF value of *CNR1* rs7766029 was 0.4806, and that of *GABRA6* rs3219151 was 0.4341 in the combined population.

## Results

### Descriptive Statistics

Mean values or frequencies of the investigated variables, and their differences between the Budapest and Manchester subsamples are displayed in Table 1. We can see that subjects from Manchester are older, more depressed and more anxious, and have experienced more stressful events related to financial and illness/personal problems within the last year than subjects from Budapest, moreover they also show differences in *CNR1* rs7766029 genotype distribution.

**Table 1:** Description of the population samples, and differences between them.

		Total sample	Cohort		
			Budapest	Manchester	Comparison (*p*-Value)
**Population size**	(N)	2193	902	1291	
**Demographics**					
Gender	(% Male)	30.10%	29.79%	30.31%	0.792
Age	(Mean ± SEM; range)	32.89 ± 0.220	31.23 ± 0.350	34.03 ± 0.279	<**0**.**0001**
		(18–60)	(18–60)	(18–60)	
**Stress type scores (within the last year)**					
RLE-relationship	(Mean ± SEM)	0.13 ± 0.008	0.13 ± 0.013	0.14 ± 0.010	0.536
RLE-financial	(Mean ± SEM)	0.23 ± 0.011	0.15 ± 0.014	0.28 ± 0.016	<**0**.**0001**
RLE-illness/personal	(Mean ± SEM)	0.37 ± 0.013	0.33 ± 0.020	0.39 ± 0.017	**0**.**028**
RLE-social	(Mean ± SEM)	0.41 ± 0.013	0.41 ± 0.021	0.40 ± 0.017	0.727
**Recent depression/anxiety**					
Current BSI depression score	(Mean ± SEM)	0.87 ± 0.019	0.57 ± 0.023	1.07 ± 0.028	<**0**.**0001**
Current BSI anxiety score	(Mean ± SEM)	0.89 ± 0.019	0.70 ± 0.023	1.01 ± 0.027	<**0**.**0001**
**Genotype**					
*CNR1* rs7766029					**0.011**
CC	(N)	594	214	380	
TC	(N)	1094	466	628	
TT	(N)	505	222	283	
MAF (minor allele)		0.48 (T)	0.50 (C)	0.46 (T)	
*GABRA6* rs3219151					0.791
CC	(N)	434	183	251	
TC	(N)	1053	424	629	
TT	(N)	719	295	424	
MAF (minor allele)		0.44 (C)	0.44 (C)	0.43 (C)	


*BSI, Brief Symptom Inventory; RLE, number of recent negative life events (within the last year); RLE-financial, financial difficulties; RLE-illness/personal, illness, and personal problems; RLE-relationship, intimate relationship problems; RLE-social, social network disturbances; SEM, standard error of mean; MAF, minor allele frequency; comparison, *p*-Value of the *t*-test or Pearson χ^*2*^ test, as appropriate. Significant *p*-values (< 0.05) are marked in bold.*

Hardy–Weinberg equilibrium did not deviate from the expected in case of either SNP (single nucleotide polymorphisms) in the combined sample or in any of the two subsamples. For *CNR1* rs7766029 *p*-values were 0.967 in the combined sample, 0.337 in Budapest, and 0.402 in Manchester populations. For *GABRA6* rs3219151, 0.115 in the combined, 0.090 in Budapest, and 0.536 in Manchester samples.

Gene-environment correlation results, calculated in additive linear regression models, are displayed in Table 2. *GABRA6* rs3219151 is significantly related to RLE-illness/personal in Budapest, and *CNR1* rs7766029 is significantly related to RLE-social in Manchester.

**Table 2:** Gene-environment correlation between *CNR1* (rs7766029) and *GABRA6* (rs3219151) polymorphisms and different types of recent negative life events.

	*CNR1* (rs7766029)				*GABRA6* (rs3219151)			
Stress type (within the last year)	β	S.E.	STAT	P	β	S.E.	STAT	P
**Combined population sample of Budapest and Manchester**					
RLE – relationship	-0.014	0.0114	-1.185	0.236	0.003	0.0113	0.244	0.808
RLE – financial	0.019	0.0156	1.191	0.234	-0.009	0.0155	-0.594	0.552
RLE – illness/personal	-0.018	0.0185	-0.973	0.331	0.004	0.0183	0.232	0.817
RLE – social	-0.025	0.0190	-1.332	0.183	0.010	0.0188	0.554	0.580
**Budapest subsample**					
RLE – relationship	-0.015	0.0185	-0.819	0.413	-0.012	0.0181	-0.661	0.509
RLE – financial	0.0002	0.0205	0.011	0.991	0.004	0.0201	0.200	0.842
RLE – illness/personal	-0.040	0.0289	-1.373	0.170	0.062	0.0283	2.205	0.028
RLE – social	0.028	0.0300	0.922	0.357	0.033	0.0291	1.127	0.260
**Manchester subsample**					
RLE – relationship	-0.014	0.0144	-0.951	0.342	0.009	0.0145	0.596	0.551
RLE – financial	0.029	0.0221	1.313	0.189	-0.027	0.0222	-1.231	0.219
RLE – illness/personal	-0.004	0.0241	-0.172	0.864	-0.039	0.0241	-1.616	0.106
RLE – social	-0.060	0.0245	-2.459	**0.014**	-0.004	0.0246	-0.169	0.866


*S.E., standard error of beta; STAT, coefficient t-statistic; RLE, number of recent negative life events (within the last year); RLE – financial, financial difficulties; RLE-illness/personal, recent illness and personal problems; RLE – relationship, intimate relationship problems; RLE – social, social network disturbances. We tested additive linear regression models. The effect allele is T in case of *CNR1* rs7766029, and C in case of *GABRA6* rs3219151. In the combined population sample of Budapest and Manchester there were no significant *p*-Values. Significant *p*-values (< 0.05) are marked in bold.*

### Primary Analyses: Interaction Effects of *CNR1* and *GABRA6* With Different Types of Recent Stress

**FIGURE 1:**
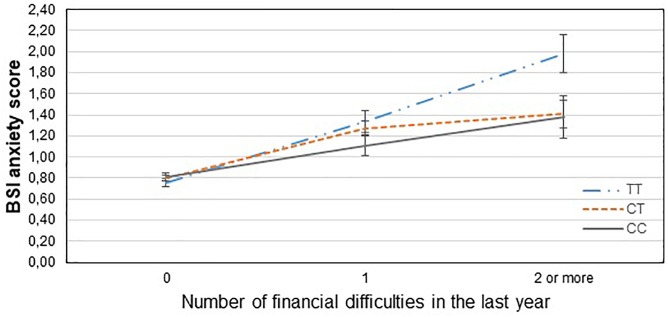
Significant interaction effect of financial difficulties and *CNR1* rs7766029 on current anxiety scores in the total sample. Mean BSI anxiety scores are displayed with standard error bars, in function of genotype and the number of life events related to financial difficulties occurred within the last year (general linear model performed only for visualization purposes).

**FIGURE 2:**
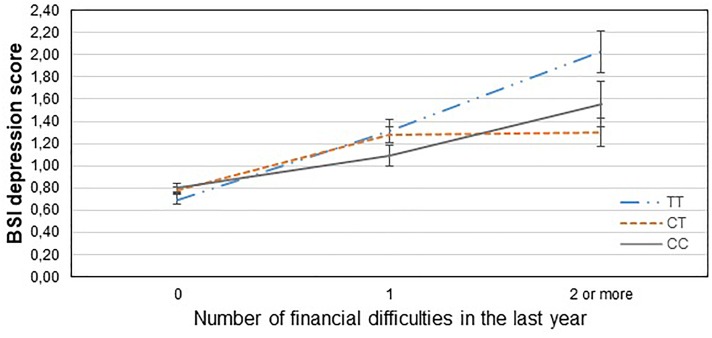
Significant interaction effect of financial difficulties and *CNR1* rs7766029 on current depression scores in the total sample. Mean BSI depression scores are displayed with standard error bars, in function of genotype and the number of life events related to financial difficulties occurred within the last year (general linear model performed only for visualization purposes).

**FIGURE 3:**
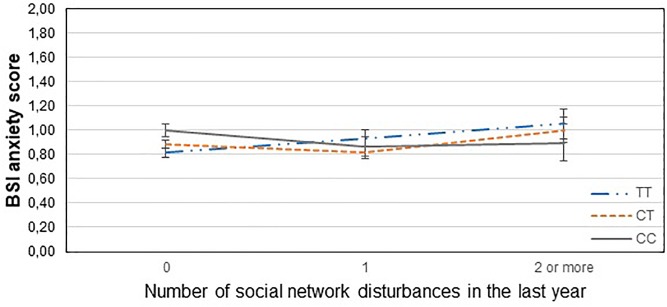
Significant interaction effect of social network disturbances and *GABRA6* rs321915 on current anxiety scores in the total sample. Mean BSI anxiety scores are displayed with standard error bars, in function of genotype and the number of life events related to social network disturbances occurred within the last year (general linear model performed only for visualization purposes).

**FIGURE 4:**
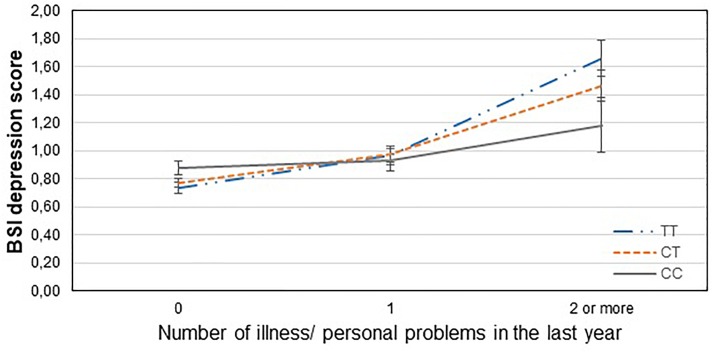
Significant interaction effect of illness/personal problems and *GABRA6* rs3219151 on current depression scores in the total sample. Mean BSI depression scores are displayed with standard error bars, in function of genotype and the number of life events related to illness, injury or problems occurred within the last year (general linear model performed only for visualization purposes).

Table 3 displays results of the 16 primary tests and shows that the interaction between *CNR1* rs7766029 and RLE-financial is significant both on BSI anxiety score (Figure 1) and BSI depression score (Figure 2). However, *GABRA6* rs3219151 exerts a significant interaction with RLE-social on BSI anxiety score (Figure 3), and with RLE-illness/personal on BSI depression score (Figure 4).

**Table 3:** Interactions of number of recent negative life events and *CNR1* (rs7766029) or *GABRA6* (rs3219151) polymorphisms on BSI anxiety and BSI depression scores as the outcome in the combined Budapest + Manchester sample.

	*CNR1* (rs7766029)					*GABRA6* (rs3219151)				
Stress type (within the last year)	β	S.E.	STAT	P	FDR Q	β	S.E.	STAT	P	FDR Q
**BSI anxiety score**					
Interaction with RLE – relationship	0.006	0.0679	0.084	0.933	0.538	-0.045	0.0720	-0.623	0.533	0.424
Interaction with RLE – financial	0.140	0.0498	2.815	**0.005**	**0.022**	-0.077	0.0506	-1.525	0.127	0.172
Interaction with RLE – illness/personal	0.007	0.0433	0.152	0.880	0.538	-0.059	0.0423	-1.392	0.164	0.172
Interaction with RLE – social	-0.007	0.0435	-0.168	0.867	0.538	-0.096	0.0415	-2.316	**0.021**	**0.045**
**BSI depression score**					
Interaction with RLE – relationship	-0.037	0.0683	-0.535	0.593	0.432	-0.100	0.0724	-1.379	0.168	0.172
Interaction with RLE – financial	0.134	0.0503	2.664	**0.008**	**0.023**	-0.057	0.0512	-1.112	0.266	0.233
Interaction with RLE – illness/personal	0.073	0.0436	1.682	0.093	0.162	-0.125	0.0426	-2.928	**0.003**	**0.022**
Interaction with RLE – social	0.001	0.0440	0.021	0.984	0.538	-0.057	0.0420	-1.349	0.177	0.172


*BSI, Brief Symptom Inventory; FDR, false discovery rate; S.E., standard error of beta; STAT, coefficient t-statistic; RLE, number of recent negative life events (within the last year); RLE – financial, financial difficulties; RLE-illness/personal, recent illness and personal problems; RLE – relationship, intimate relationship problems; RLE – social, social network disturbances. We tested additive models on a combined population sample of Budapest and Manchester. The effect allele is T in case of *CNR1* rs7766029, and C in case of *GABRA6* rs3219151. All significant *P*-values (marked in bold) survived correction for multiple testing (*P* ≤ 0.05, FDR *Q* ≤ 0.05).*

### Secondary Analyses: Replicability ofSignificant Results in the TwoSubsamples

Table 4 shows replicability of significant primary results within the separate Budapest and Manchester subsamples. None of the results proven significant in the combined sample could be replicated in both of the subsamples. However, the interaction of *GABRA6* rs3219151 and RLE-illness/personal is significant in Manchester and a trend in Budapest despite the differences in subpopulation characteristics demonstrated in Table 1, suggesting a rather generalizable effect. Nevertheless, *GABRA6* rs3219151 has shown a gene-environment correlation with RLE-illness/personal in Budapest (Table 2), albeit with the opposite direction of effect compared to that of the GxE on BSI depression (Tables 3, 4).

## Discussion

In our present study we demonstrated that variants in *GABRA6* and *CNR1* genes, previously implicated in both stress and depression (Juhasz et al., 2009, 2017; Gonda et al., 2017), interact with particular types of recent life stressors in influencing depression and anxiety. These results suggest that different genes and neurochemical systems may mediate the effects of different types of recent stress. Furthermore, while stress is a significant factor associated with both depression and anxiety, which overlap significantly both genotypically and phenotypically, we found that the two investigated genetic variants interacted with different types of stress in the background of these phenotypes. Specifically, in case of anxiety *CNR1* rs7766029 showed an interaction with recent financial stress and *GABRA6* rs3219151 with recent social network-related stressors. In case of depression, *CNR1* rs7766029 similarly interacted with recent financial stress, *GABRA6* rs3219151, however, interacted with recent illness and personal problem stressors. Our findings, thus, also show that while certain genes uniformly mediate the effect of a given type of recent stressor in the development of various facets of psychopathology, others may transform different stressors into distinct phenotypes.

**Table 4:** Re-analysis of data split according to study site in the Budapest and Manchester subsamples.

	Budapest subsample				Manchester subsample			
Gene-environment interaction	β	S.E.	STAT	P	β	S.E.	STAT	P
**BSI anxiety score**					
*CNR1* interaction with RLE – financial	0.127	0.0794	1.601	0.110	0.143	0.0652	2.188	**0.029**
*GABRA6* interaction with RLE – social	-0.067	0.0501	-1.338	0.181	-0.111	0.0622	-1.781	*0.075*
**BSI depression score**					
*CNR1* interaction with RLE – financial	0.061	0.0797	0.768	0.443	0.163	0.0660	2.464	**0.014**
*GABRA6* interaction with RLE – illness/personal	-0.087	0.0514	-1.695	*0.090*	-0.144	0.0633	-2.280	**0.023**


*BSI, Brief Symptom Inventory; S.E., standard error of beta; STAT, coefficient t-statistic; RLE, number of recent negative life events (within the last year); RLE – financial, financial difficulties; RLE-illness/personal, recent illness and personal problems; RLE – social, social network disturbances. We tested replicability of the interactions in the Budapest and Manchester subsamples. The effect allele is T in case of *CNR1* rs7766029, and C in case of *GABRA6* rs3219151. Significant *P*-values are marked in bold, and trends are marked in italic. No correction for multiple testing was applied.*

### The Role of the Endocannabinoid System and the CB1 Receptor in Stress, Depression, and Anxiety

Modulation of the stress response and stress adaptation is one of the major roles and effects of the central nervous endocannabinoid system (Patel and Hillard, 2008). The presynaptic CB1 endocannabinoid receptors with dense localizations in the prefrontal cortex, amygdala and hippocampus (Morena and Campolongo, 2014) play crucial roles in this process (McLaughlin et al., 2014). The two retrogradely acting endocannabinoids, anandamide (AEA) and 2-arachidonoylglycerol (2-AG), modulate glutamate and GABA neurons following their release and, thereby, balance excitatory and inhibitory activity (Freund et al., 2003; Hill et al., 2010; McLaughlin et al., 2014). CB1 activation also enhances brainstem serotonin and noradrenaline activity and regulates the sensitivity and activity of the hypothalamus-pituitary-adrenal (HPA) axis in stress adaptation and habituation (Gorzalka et al., 2008; Steiner and Wotjak, 2008; Hill et al., 2010; McLaughlin et al., 2014). While decreased CB1 transmission contributes to a state resembling chronic stress (Lazary et al., 2011), chronic stress alters endocannabinoid transmission contributing to stress habituation (Patel and Hillard, 2008).

The role of the endocannabinoid system and the CB1 receptor in depression and anxiety is also supported by several lines of animal studies linking reduced CB1 signaling to increased depression and anxiety (Hill and Gorzalka, 2005). CB1 KO mice showed increased sensitivity toward developing learned helplessness, an animal model of depression, as well as anhedonia upon exposure to chronic mild stress (Gorzalka et al., 2008), and CB1 receptor mediated endocannabinoid transmission was found to play a role in influencing affective processing under stress exposure in both rodents (McLaughlin et al., 2012; Wang et al., 2012; Morena et al., 2016) and humans (Wirz et al., 2018). In addition, the importance of the cannabinoid signaling in mood disorders is specifically highlighted by the severe increase in depressive and anxiety symptoms following administration of the CB1 antagonist appetite suppressant rimonabant in previously non-depressed human subjects (Christensen et al., 2008; Goodwin et al., 2012). Such studies suggest that the endocannabinoid system buffers the emotional effects of stress exposure (Morena and Campolongo, 2014; Morena et al., 2016), and raise the possibility of variations in the *CNR1* gene (encoding the CB1 receptor) altering the neural information processing of affective stimuli (Wirz et al., 2018). The endocannabinoid system has also been reported to play a role in stress-induced anxiety, with pharmacological or genetic disruption of CB1 signaling increasing anxiety moderately without, and dramatically following stress exposure (Haller et al., 2004; Hill et al., 2011; Morena et al., 2016). Indeed, in humans disrupted CB1 signaling related to *CNR1* variation appears to be involved in stress vulnerability and manifestation of stress related psychiatric conditions (Morena et al., 2016) including schizophrenia (Fernandez-Espejo et al., 2009; Gouvea et al., 2017) as well as non-response to antipsychotics; substance abuse and addiction (Benyamina et al., 2011; Lopez-Moreno et al., 2012); eating disorders; autism (Hillard et al., 2012); and mood and anxiety disorders (Ashton and Moore, 2011; Hillard et al., 2012).

### *CNR1* rs7766029 Selectively Mediates the Effects of Financial but Not Other Types of Recent Stress on Depression and Anxiety Symptoms

The *CNR1* gene encoding the human endocannabinoid receptor 1 (CB1) is located on chromosome 6q14-15 and while the function of SNPs in the *CNR1* gene and their effect on expression and activity has not yet been reported, they may decrease mRNA stability and thus contribute to reduced CB1 receptor expression (Domschke et al., 2008). A significant association was reported between *CNR1* and high neuroticism, which leads to a propensity to both perceive and experience life events as more negative and stressful as well as to less adaptive coping in the face of stress further stressing the role of this variant in the emergence of stress-related psychopathology (Juhasz et al., 2009). *CNR1* variation increases risk and vulnerability to depression upon exposure to both early and recent stress (Juhasz et al., 2009; Agrawal et al., 2012). In particular, the 3^′^-end polymorphism rs7766029 has been suggested to play a role in the development of depression by impacting the experience of negative life events through increased exposure by life choice or through response to or interpretation of negative life events (Bouchard and Mcgue, 2003; Juhasz et al., 2009; Aleksandrova et al., 2012) but it remained uninvestigated whether this gene uniformly mediates the effects of all types of stressors. Thus this is the first study to report that *CNR1* rs7766029 selectively mediates the effects of financial stress, but not other types of recent life events in both depression and anxiety symptoms.

Similarly to our present results, we previously reported that in a general population sample, in males but not in females, and in those above, but not under thirty years of age, that the *5-HTTLPR* polymorphism of the serotonin transporter gene mediated the effects of recent financial but not other types of stressors in the background of depression (Gonda et al., 2016a,b). Variation in the neural nitric oxide synthase (*NOS I*) gene also interacted with financial difficulty in the development of depression in another study (Sarginson et al., 2014). In addition, financial stress was found to predict both risk and persistence of depression and also associated with lower remission rates during antidepressant treatment with citalopram (Trivedi et al., 2006). It has also been previously reported that early childhood financial difficulties show a strong association with reduced connectivity in the default mode network, which remains observable in adulthood and it associated with increased stress sensitivity based on increased cortisol production during social stress anticipation (Sripada et al., 2014). This suggests a distinct effect of financial problems versus other types of stressors. Financial stress can arguably be viewed as a proxy for a threat for general safety of existence and thus can be considered a pervasive, and especially in extreme cases a severe and life-threatening stressor as opposed to other, non-existential types of environmental and life events. The importance of such stress and the vital threat linked with it may be one reason why the effect of this type of recent stress is mediated by multiple genes and neurochemical systems and argues for its strong role in the background of various manifestations of stress-related psychopathologies.

### The Role of the GABA System and *GABRA6* Variation in Stress, Depression, and Anxiety

The GABA system plays an important role in acute stress reaction (Giordano et al., 2008) while stress exposure also exerts both short and long-term effects on GABA signaling, including changes in composition, sensitivity and availability of GABA-A receptors which in turn contribute to alterations in the stress response (Skilbeck et al., 2010; Gunn et al., 2011; Luscher et al., 2011). The GABA system plays a key role in the HPA-axis downregulation in response to stress as demonstrated by the strong inhibitory effect on the HPA axis by alprazolam, an anxiolytic agent enhancing GABA signaling by increasing the affinity of GABA for the GABA-A receptor (Giordano et al., 2006). Studies have shown that the GABA system in interaction with stress influences central nervous stress control through GABA-A receptors localized on hypothalamic CRH neurons in the paraventricular nuclei, inhibiting the HPA axis (Oquendo and Mann, 2000; Luscher et al., 2011) and attenuating response to stress (Gunn et al., 2011). Chronic stress, however, leads to reduced GABA activity and thus altered response during subsequent stress exposure (Hu et al., 2010; Gunn et al., 2011; Luscher et al., 2011).

Disrupted GABA signaling and GABA deficit is also hypothesized to play a role in the development of depression and anxiety (Luscher et al., 2011), while Gad2 mRNA, the synthesis enzyme of GABA, was upregulated after chronic treatment with the serotonin and noradrenaline reuptake inhibitor venlafaxine (Tamasi et al., 2015). Reciprocally, the GABA system is also involved in regulating and fine-tuning depression-relevant serotonergic and noradrenergic processes (Luscher et al., 2011; Pehrson and Sanchez, 2015). Furthermore, it appears that decreased HPA axis inhibition possibly related to GABA-A receptor variation may in turn lead to increased physiological stress response and higher risk of mental health disorders like depression (Uhart et al., 2004). These results again point to the possible involvement of GABA-A receptor variations in the modulation of stressful stimuli in psychiatric phenotypes, nevertheless, studies failed to investigate the roles related to specific stressors.

### *GABRA6* rs3219151 Shows a Divergent Pattern in Mediating Different Types of Recent Stressors in the Background of Depression and Anxiety

Rs3219151, located in the 3^′^ untranslated region in the GABRA6 gene encoding the alpha-6 subunit of the GABA-A receptor, and predicted to be in the target region of 4 microRNAs, has previously been demonstrated to play a significant role in modulating HPA-axis activity. Those carrying the T allele exhibited higher plasma cortisol levels both during resting conditions (Rosmond et al., 2002) as well as during stimulation in the Trier Social Stress Test (Uhart et al., 2004), showing that this allele is, indeed, associated with increased stress response. Other studies similarly reported association between GABA-A subunit variation and stress reactivity manifested in increased blood pressure, cortisol, and adrenocorticotropic hormone (ACTH) levels following stress in *GABRA6* rs3219151 T allele carriers (Sen et al., 2004; Uhart et al., 2004; Inoue et al., 2015). In a recent study we found that while the T allele of *GABRA6* rs3219151 was not directly associated with either depression or anxiety, a strong effect was observed in interaction with recent life stress on both anxiety and depression (Gonda et al., 2017).

In our present study we observed that *GABRA6* rs3219151 mediated the effects of different types of life events in the background of depression and anxiety. While in case of depression the effects of rs3219151 were observable in case of exposure to recent illness- and personal problem related life events, in case of anxiety rs3219151 only interacted with recent social-network related stressors. In both cases, just as in case of our previous study (Gonda et al., 2017), presence of the T allele increased risk of depression and anxiety. This novel finding corroborates previous reporting that those carrying the T allele show a larger stress reactivity and expand these results with the information that this increased reactivity appears to be specific to certain life events.

In line with our previous study of 5-HTTLPR (Gonda et al., 2016a), the present results of *CNR1* rs7766029 support a distinct role of financial stress among different life events in the development of depression and anxiety symptoms possibly related to the prominent and pervasive impact of this type of stress. However, our findings concerning *GABRA6* rs3219151 indicate that other types of stressors may also be selectively mediated by distinct genetic elements, suggesting that the effects of different types of hardships are mediated by distinct neurochemical pathways. Furthermore, in case of *GABRA6* rs3219151 we also observed that different types of recent stressors contribute to the emergence of different types of psychopathology: depress ion in case of illness and personal concerns and anxiety in case of social network-related problems. A further interesting aspect of these results is that in case of anxiety, a crossover pattern was observed with the “risk” T allele associated with lower anxiety without stress exposure and a higher anxiety in those exposed to severe stress. This is in part similar to a previous finding of *5-HTTLPR* in a population aged younger than 30 years, we similarly found a crossover pattern in interaction with recent social-network related stress, however, in that case the “risk” s allele of *5-HTTLPR* was associated with decreased depression when exposed to severe stress (Gonda et al., 2018a). Thus, our present findings also show that the same type of stressor may increase the risk or protect against different types of psychological symptoms depending on the interacting gene and the involved neurochemical system. Which, in part, may explain for the presence of these variations in the genome and the evolutionary maintenance of pro-depressive states.

### The Importance of Investigating the Type of Life Events in Gene × Environment Interactions

The divergent genetic interaction patterns of distinct types of recent stress in the background of depression and anxiety has not been widely investigated so far, although in case of childhood traumas the differential genetic interaction effects of different types of maltreatment is already well-known (Cicchetti et al., 2007; Fisher et al., 2013). Our present results clearly show that recent stress is a heterogeneous phenomenon and different types of recent life events and stressors may exert their effect via different neurobiological pathways and mechanisms in the emergence of depression and anxiety. These findings also expand our previous results where we found that genes belonging to different neurochemical pathways mediate the effects of moderate or severe stress in depression, with some genetic variants being more relevant in case of moderate and others in case of severe recent stress exposure (Gonda et al., 2018b). In spite of these, there is little attention paid to subcategorization of life events according to type and severity in genetic studies of depression, and lack of consideration of the distinct genetic and biochemical pathways of different stressors may be an important contributor to the lack of positive findings and replicability in gene-environment interaction studies in depression (Gonda et al., 2018c). Furthermore if certain genetic variants increase risk of depression or anxiety only when exposed to specific stressors and certain stressors only lead to depression and anxiety in those carrying given genetic variants, this may contribute to a more sophisticated understanding of predicting, screening and preventing depression. Such findings may not only advance our understanding on the complex pattern of interaction between stressors and genes, but could also help in the refinement of subtyping affective disorders, pinpointing new pharmacological targets and advancing precision treatment of these illnesses.

### Limitations

There are several limitations of our present study. First of all, we would emphasize the exploratory nature of our analyses and urge replication of the results presented here in other cohorts. Second, our research is cross-sectional, thus it is possible that depressive symptoms developing with a greater latency following exposure or repeated exposure remained hidden. Third, recent life events occurring in the previous year were recorded retrospectively, and these, just as measures of depression and anxiety were based on self-report. Fourth, recall of negative life events is subject to recall bias, which is, on the one hand memory dependent, and on the other hand, state dependent, so it is possible that those less depressed recalled less, while those more depressed recalled more negative life events. Fifth, our study sample was a general population sample of volunteers, which contributes to a possibility of sampling bias with respect to depression. Sixth, we used two geographically different subsamples in our study, and ancestry was not assessed in the present study using molecular methods such as whole-genome SNP genotyping. Although to consider this we used population as a covariate in all our statistical analyses, there may exist subtle genetic differences both between the two subsamples and also within each sample due to population stratification which may lead to spurious effects. Finally, while our reported results were significant in the combined population, we could not replicate some of them separately in the two population subsamples.

## Conclusion

In our study we demonstrated that genetic variation in two distinct neurochemical systems, namely, rs7766029 in the *CNR1* and rs3219151 in *GABRA6*, mediate the effects of different types of recent stress in depression or anxiety. Interestingly, in case of *GABRA6*, the resulting psychological symptom may be the function of the type of stress experienced. Our findings thus show that stress is a heterogeneous phenomenon and different types of it may activate different neural pathways. Besides its possible clinical and pharmacological implications, our results suggest that the environmental context of psychiatric symptoms, disorders and relevant genes should be specified in a more detailed and multidimensional manner in order to be able to better predict, prevent and treat affective illnesses.

## Ethics Statement

This study was carried out in accordance with the Declarations of Helsinki. All subjects gave written informed consent and the study protocol was approved both by the Scientific and Research Ethics Committee of the Medical Research Council, Budapest, Hungary, and the North Manchester Local Research Ethics Committee, Manchester, United Kingdom.

## Author Contributions

XG, PP, IA, BD, GJ, and GB conceived and designed the study. XG, PP, NE, and GJ participated in recruiting and evaluating the study sample and collecting the DNA samples. NE, SS, ZG, and SK participated in managing and analyzing the data. XG, PP, NE, SS, ZG, SK, IA, BD, GJ, and GB participated in interpreting the data. XG and PP wrote the first draft of the manuscript. All authors revised subsequent versions of the manuscript, contributed to and approved the final version of the manuscript.

## Conflict of Interest Statement

BD variously performed consultancy, speaking engagements and research for P1vital, Autifony, and AstraZeneca; fees are paid to The University of Manchester; he has share options in P1vital. IA has received consultancy fees from Servier, Alkermes, Lundbeck/Otsuka, and Janssen, an honorarium for speaking from Lundbeck and grant support from Servier and AstraZeneca. The remaining authors declare that the research was conducted in the absence of any commercial or financial relationships that could be construed as a potential conflict of interest.

## Footnotes

1 www.sequenom.com

2 https://www.cog-genomics.org/plink2

3 http://biostats.usc.edu/Quanto.html
